# Comparative Effects of Zinc and Probiotics on Neonatal Indirect Hyperbilirubinemia Undergoing Phototherapy

**DOI:** 10.7759/cureus.104124

**Published:** 2026-02-23

**Authors:** Malik Muhammad Umair Fazal, Muhammad Anwar, Muhammad Umar Shafiq, Rabia Yousaf, Muhammad Shahid, Sumaira Abbasi

**Affiliations:** 1 Department of Neonatology, Quaid-e-Azam Medical College, Bahawalpur, PAK; 2 Department of Pediatrics, Quaid-e-Azam Medical College, Bahawalpur, PAK

**Keywords:** hyperbilirubinemia, jaundice, neonate, probiotics, zinc

## Abstract

Background

Neonatal indirect hyperbilirubinemia remains a frequent cause of admission to neonatal units in Pakistan. Although phototherapy is effective, prolonged treatment increases hospital stay, parental anxiety, and healthcare burden. Adjunctive therapies such as zinc and probiotics have been evaluated to enhance bilirubin reduction, but comparative local evidence is limited and inconsistent.

Objectives

To compare the effects of zinc and probiotics as adjuncts to phototherapy on bilirubin decline and selected clinical outcomes in neonates with indirect hyperbilirubinemia.

Methods

A single-center, parallel-group randomized controlled trial was conducted in the Department of Neonatology, Quaid-e-Azam Medical College, Bahawalpur, for six months. A total of 130 neonates requiring phototherapy were randomized equally to receive either oral zinc (5 mg once daily) or oral probiotics (1×10⁹ colony-forming units once daily). This was done in addition to standard phototherapy. The primary outcome was the rate of decline in total serum bilirubin. Secondary outcomes included the duration of phototherapy, length of hospital stay, rebound hyperbilirubinemia, and adverse events.

Results

Baseline demographic and clinical characteristics were comparable between groups. A higher mean bilirubin reduction at 24 hours was observed in the zinc group compared with the probiotic group. A greater proportion of neonates receiving zinc achieved a bilirubin decline of at least 0.2 mg/dL per hour. The median duration of phototherapy and length of hospital stay were shorter in the zinc group. Rebound hyperbilirubinemia and adverse events were infrequent and did not differ significantly between groups. No exchange transfusion was required.

Conclusions

Bilirubin decrease was faster, and the duration of phototherapy was shorter when zinc supplements were added than when probiotics were taken. The findings of these observations support the possible usefulness of zinc in the context of neonatal indirect hyperbilirubinemia management in low-resource environments.

## Introduction

Indirect hyperbilirubinemia in newborns is still one of the most common etiologies of premature admission into hospitals. Neonatal jaundice is still found to be a huge burden on the overstretched neonatal services in Pakistan. It is estimated that more than half of term neonates develop clinically apparent jaundice, and a substantial percentage of affected neonates need phototherapy due to increasing bilirubin levels [1]. Outcomes may be aggravated by late presentation, lack of recognition at home, and inadequate follow-up facilities. Serious hyperbilirubinemia is an avoidable cause of neurological dysfunction in poor-resource countries like Pakistan, where kernicterus has never been completely eliminated [2].

As a result of more bilirubin production and immaturity of hepatic conjugation, indirect hyperbilirubinemia occurs. The enzyme uridine diphosphate glucuronosyltransferase of neonates is functionally immature and leads to the accumulation of non-conjugated bilirubin in the blood. They play a major role in enterohepatic circulation. Increased intestinal reabsorption of bilirubin may also increase serum levels. Premature births, low birth weights, hemolysis, feeding, dehydration, and sepsis are among the risk factors [3]. Without treatment, elevated bilirubin levels can cross through the blood-brain barrier and result in bilirubin-related neurological dysfunction. Acute bilirubin encephalopathy and kernicterus [4] are chiefly seen as a consequence of untreated bilirubin.

Neonatal jaundice is one of the main causes of neonatal morbidity in Asia. South Asian and Southeast Asian studies have higher rates of severe hyperbilirubinemia relative to high-income regions [5]. Phototherapy is the most common form of therapy in most Asian environments. However, long-term phototherapy causes a rise in both hospitalization and healthcare expenditures. Therefore, adjunctive bilirubin-lowering therapies are under preclinical research. It was also proposed that zinc might decrease enterohepatic circulation by binding with unconjugated bilirubin in the intestine, and probiotics could alter gut flora and decrease beta-glucuronidase, and thus reduce bilirubin reabsorption [6].

There has been an increase in global interest in adjuncts to phototherapy in non-invasive forms. According to a randomized trial of South African origin, oral zinc supplementation can be used to reduce phototherapy time in neonates with indirect hyperbilirubinemia [7]. A study among North Americans showed mixed results and did not indicate a strong advantage of zinc in improving the decline in bilirubin but raised a question of population-specific effects [8]. Another Canadian study also highlighted the possible effect of gut microbiota regulation on the metabolism of bilirubin in early life that establishes a theoretical role of probiotics [9]. However, in a Chinese randomized clinical trial, quicker bilirubin decline was observed. A shorter phototherapy span was also observed in infants administered probiotics on top of conventional hospital treatment [10]. Such heterogeneous results are open to differences in feeding habits, baseline zinc status, and patterns of gut colonization of the newborn.

Data on Pakistan are limited despite increasing international evidence. Nutritional zinc deficiency is common in pregnant women and neonates in this part of the world. This is probably because of neglected antepartum and postpartum care in a large portion of women and babies born in rural areas of the country. The local population in Pakistan is also characterized by breastfeeding habits, late passage of meconium, and increased cases of neonatal infections. Therefore, results from high-income countries may not be directly generalizable to Pakistani neonates. Moreover, most available studies have evaluated either zinc or probiotics separately. A direct comparative evidence between these two interventions is limited, particularly in low-resource neonatal units.

The primary objective of this study was to compare the effects of zinc versus probiotics as adjuncts to phototherapy on bilirubin reduction and treatment duration in neonates with indirect hyperbilirubinemia. This trial does not include a phototherapy-only arm, and the comparison is only between zinc and probiotics as adjuncts to phototherapy. The primary endpoint was the absolute bilirubin reduction at 24 hours, with secondary outcomes including the rate of bilirubin decline, duration of phototherapy, and length of hospital stay.

## Materials and methods

This study was designed as a single-center, parallel-group, randomized controlled trial, which utilized a superiority hypothesis. Randomization was undertaken at a 1:1 ratio. The trial was conducted at the Department of Neonatology in Quaid-e-Azam Medical College and its affiliate teaching hospital in Bahawalpur. It lasted six months, starting with the commencement of the trial on July 15, 2025, and ending on January 15, 2026. All involved neonates were observed prospectively from the initiation of phototherapy until they were discharged from the neonatal unit or until they reached their treatment goal, whichever came sooner.

The ethical committee approved the study (approval number ERC 248/DME/QAMC Bahawalpur), dated July 7, 2025. The research was listed on the ClinicalTrials.gov site (NCTID: 07102836) on July 30, 2025 before enrolment.

The eligible subjects were neonates presenting with indirect hyperbilirubinemia requiring phototherapy. Inclusion criteria included infants of either sex, gestational age of 35 or more weeks, a postnatal age of 14 days or less, and a diagnosis of unconjugated hyperbilirubinemia based on serum bilirubin levels. Phototherapy was indicated based on current clinical practice. Exclusion criteria included conjugated hyperbilirubinemia, major malformations at birth, suspected or confirmed inborn errors of metabolism, previous exchange transfusion, severe birth asphyxia, severe malformation of the gastrointestinal tract, and any infant that had received zinc or probiotic supplements prior to admission.

The two-proportion comparison framework was applied in establishing the size of the sample required in this randomized controlled trial. The calculation was based on a previous randomized, placebo-controlled trial on the effect of zinc supplementation in neonates with indirect hyperbilirubinemia with statistically significant better bilirubin-related outcomes [11]. In the same study, approximately 60% of infants in the control arm needed protracted phototherapy, but only about 35% of infants in the zinc-enriched study needed protracted treatment, a decrease in absolute risk by 25%.

Using these anticipated proportions, the traditional two-proportion sample-size equation was used. A power of 80% and alpha (two-sided) of 0.05 were used. The corresponding Z-values were 1.96 (alpha) and 0.84 (power). These parameters provided a sample requirement of 59 neonates per arm. The nominal sample size was set to 65 participants per group to cover an estimated attrition (approximately 10%). Thus, the number of infants was 130, which had sufficient power to identify a statistically significant effect between the intervention groups.

The eligible infants were recruited to participate in the study in the neonatal ward where they were screened by the duty neonatology nurses. Written informed consent was obtained before enrolment from parents or legal guardians. The discussions about consent were conducted in the local language, ensuring that they understood them properly. Enough time was taken to allow the respondent to ask questions prior to consent being given.

After enrolment, the participants were randomly assigned to two intervention arms. One group was given oral supplementation of zinc on top of the regular form of phototherapy, and the other was given oral probiotics along with the usual form of phototherapy.

Phototherapy was administered using calibrated phototherapy units, specifically with blue light (wavelength range: 450-490 nm). The irradiance range of the phototherapy units was 12-15 μW/cm²/nm as per the manufacturer's specifications. The distance between the neonates and the phototherapy unit was maintained at 40 cm, in line with recommended protocols to ensure optimal efficacy. The phototherapy units were regularly checked and calibrated during the study to ensure that the irradiance was consistent and within the specified range.

Feeding practices were standardized, with neonates receiving either exclusive breastfeeding, mixed feeding, or formula feeding. These feeding types were equally distributed across the study groups to ensure no bias in the nutritional intake that could influence enterohepatic circulation.

The primary endpoint of the study was the absolute bilirubin reduction at 24 hours, defined as the change in total serum bilirubin (TSB) levels from baseline to 24 hours. A secondary endpoint was the rate of bilirubin decline (mg/dL/h) over the first 24 hours.

Both groups received identical phototherapy conditions, with the only intervention being the addition of either zinc or probiotics. There were no differences in the phototherapy beds used for the two groups.

Prolonged phototherapy was defined as a duration of phototherapy greater than 72 hours, based on clinical guidelines for neonatal hyperbilirubinemia treatment. Rebound hyperbilirubinemia was defined as a rise in total serum bilirubin to a level that requires re-initiation of phototherapy after its discontinuation, occurring within 48 hours post-phototherapy cessation, with a total serum bilirubin (TSB) level exceeding the threshold for treatment (e.g., ≥15 mg/dL).

Zinc supplementation was administered orally at a dose of 5 mg elemental zinc once daily. The zinc preparation was provided in syrup form (zinc sulfate syrup), commercially available with the name of Zincate OD suspension (Atco Laboratories, Karachi, Pakistan), and administered using a calibrated oral syringe. Probiotic supplementation was administered orally at a dose of 1×10⁹ colony-forming units once daily. The probiotic formulation contained *Lactobacillus rhamnosus* GG (commercial name: PrePro GG Drops, Matrix Pharma, Karachi, Pakistan), and was given as 10 drops (0.33 mL) per day. Both supplements were initiated within 12 hours of commencement of phototherapy and continued daily until discontinuation of phototherapy or for a maximum duration of five days, whichever occurred first.

All doses were administered by trained nursing staff. The exact time and completion of each dose were documented on a standardized treatment chart. If vomiting or feeding intolerance occurred within 30 minutes of administration, the dose was withheld and documented, and no repeat dose was given on the same day. During the treatment period, there was no dose modification. Normative neonatal care, including feeding and hydration, was upheld correctly in both groups throughout the intervention period.

The two interventions were initiated no later than 12 hours after initiation of phototherapy and continued until phototherapy was discontinued or up to a preset limit. All the participants were administered phototherapy of the regular kind using the calibrated phototherapy units in accordance with departmental procedures. The styles of feeding, hydration, and regular neonatal care were adhered to equally in both groups. No extra anti-jaundice medicine treatment was approved throughout the research.

Phototherapy initiation and discontinuation were based on the American Academy of Pediatrics (AAP) 2004 guidelines for the management of hyperbilirubinemia in newborns. The decision to start phototherapy was made when the total serum bilirubin (TSB) level exceeded the threshold for phototherapy based on the infant's age in hours and risk factors (as defined by the AAP guidelines). Phototherapy was discontinued once the bilirubin levels dropped to a safe threshold, typically below 12 mg/dL, and when the neonate was clinically stable. "Safe bilirubin levels" were defined as a TSB of less than 12 mg/dL, as recommended by the AAP guidelines for neonates in the first 24-48 hours of life. Once the bilirubin level fell below this threshold, phototherapy was discontinued unless clinically indicated otherwise.

The rate of decrease in TSB during phototherapy was set as the main outcome. The secondary outcome measures included the overall time of phototherapy, time spent in the hospital, time to safe bilirubin levels, escalation of care that may include exchange transfusion, and morbidity of rebound hyperbilirubinemia. Baseline levels of serum bilirubin and predetermined intervals of serum bilirubin levels during treatment were measured with the help of standardized laboratory methods. Any adverse medical experience that was plausibly linked with the intervention and took place at some time was classified as an adverse event. It was recorded in a systematic way.

Randomization was performed using a computer-generated random sequence. A block randomization method with a block size of four was used to ensure balanced allocation between the two groups. The randomization was performed in a 1:1 ratio, with no stratification applied based on baseline characteristics.

Participants were enrolled by trained neonatology nurses who were not involved in the randomization process. The randomization sequence was generated by an independent statistician who had no involvement in patient enrollment or treatment allocation. The allocation list was securely stored and only accessible to the statistician responsible for generating the sequence.

**Figure 1:**
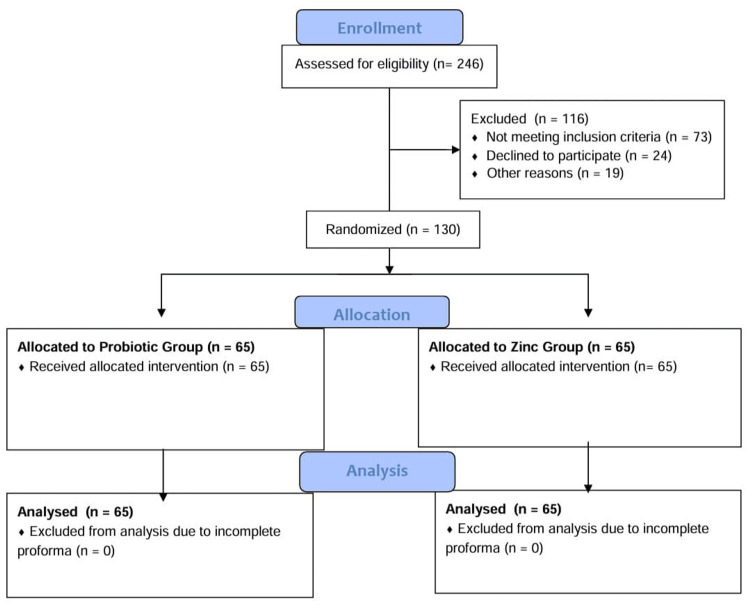
CONSORT 2010 flow diagram of the included patients CONSORT: Consolidated Standards of Reporting Trials.

To ensure allocation concealment, opaque, sealed envelopes containing the group assignments were prepared in advance. The envelopes were opened by a separate, independent research assistant at the time of enrollment, ensuring that the study personnel responsible for participant enrollment were blinded to the group assignments. The Consolidated Standards of Reporting Trials (CONSORT) diagram of participant randomization is provided in Figure 1.

A structured proforma that was created with regard to the study was used to gather data. The data were collected in clinical and laboratory form using prospectively trained research personnel. Quality checks were enforced by frequent cross-checking and monitoring. Deviations from the protocol were recorded. The analysis of the participants occurred in the groups in which they were initially assigned. Gaps in the data were addressed through a set of predetermined approaches. Missing records were omitted in particular analyses where necessary.

Statistical analysis was performed using the Statistical Package for the Social Sciences (SPSS) software, version 26 (IBM Corp, Armonk, NY). An intention-to-treat approach was adopted. Continuous variables were assessed for normality using visual inspection of histograms and the Shapiro-Wilk test. Normally distributed continuous variables, including postnatal age at admission and baseline total serum bilirubin levels, were summarized as mean±standard deviation and compared between groups using the independent samples t-test. Non-normally distributed continuous variables, including duration of phototherapy and length of hospital stay, were summarized as median with interquartile range and compared using the Mann-Whitney U test.

Categorical variables, including sex distribution, gestational age category, birth weight category, early onset of jaundice (≤48 hours), blood group incompatibility, direct Coombs test positivity, achievement of bilirubin decline ≥0.2 mg/dL per hour, prolonged phototherapy (>72 hours), rebound hyperbilirubinemia, and adverse events, were compared between groups using the chi-square test or Fisher’s exact test where appropriate.

The primary outcome, mean reduction in total serum bilirubin at 24 hours, was analyzed using the independent samples t-test. Effect sizes were expressed as mean differences or relative risks with 95% confidence intervals. A two-sided p-value of less than 0.05 was considered statistically significant.

During the study, ethical principles described in the Declaration of Helsinki were used. Participant data were confidential. Monitoring of adverse events was done all through. This was reported to the ethics committee as per the institutional policy. There were no interim analyses that would be conducted. In order to ascertain scientific rigor and participant safety, ethics approval, trial registration, informed consent, and safety monitoring were put in place.

## Results

One hundred and thirty neonates were recruited in the research period. All registered study participants were randomized and qualified on a 1:1 basis, with 65 neonates assigned to the zinc supplementation group and 65 to the probiotic group. None of the subjects was excluded after randomization. All the neonates had follow-up data until the end of phototherapy or discharge.

The primary outcome, absolute bilirubin reduction at 24 hours, showed a significant difference between the two groups (p=0.005), with a greater mean reduction in the zinc group (3.4±1.1 mg/dL) compared to the probiotic group (2.8±1.2 mg/dL). The rate of bilirubin decline over the first 24 hours was also higher in the zinc group (0.14 mg/dL/h) compared to the probiotic group (0.12 mg/dL/h).

Additionally, the median duration of phototherapy was significantly shorter in the zinc group, at 48 hours (interquartile range (IQR): 36-60), compared to the probiotic group, which had a median duration of 60 hours (IQR: 48-72), with a p-value of 0.012.

The random assignment involved 130 neonates in a 1:1 ratio in the zinc and probiotics supplementation groups. Baseline demographics and baseline clinical features were equally proportioned. Clinically significant differences were not observed with regard to sex distribution, gestational age, and birth weights or baseline bilirubin levels. Most of the neonates were term and admitted in the first week of life. This was also consistent with the normal admission trends in neonatal units.

There was a pattern of the general outcome. The percentage of the rate of decrease in bilirubin was higher in the zinc group compared to the probiotic group. The proportion of neonates who reached a faster decline threshold was greater in the zinc group. This was statistically significant. The pattern was consistent within the gestational age and categories contributed during feeding. This was despite having very low formal sub-analysis.

The duration of phototherapy represented one of the notable secondary outcomes that were affected by the difference. Neonates who were given the zinc supplementation took less time under the phototherapy. Cases of delayed phototherapy (over 72 hours) were also low in this category. Hospital stay was also similar. Shorter stays occurred in the case of the neonates who were given zinc.

There were a few variables that did not show any signs of differences between the groups. Baseline total serum bilirubin, peak category bilirubin, blood group incompatibility, positive direct Coombs test, and hemolysis markers were also distributed equally. Rebound hyperbilirubinemia was rather uncommon and no longer significant between groups. Further to show general clinical wishes in the observation group, no citizen who was put under neonatal exchange experienced a subsequent exchange transfusion.

There was mild variation of feeding patterns and stool frequency between the groups. The probiotic group had a higher orthologue of the stool frequency. This was not statistically significant. The exposure to antibiotics and screening of sepsis were low and equal. The side effect was mild, and gastrointestinal symptoms were reported more often in the probiotic group. The intervention was not terminated because of any adverse effects.

Overall, the observed results showed a mix of statistically significant and non-significant findings. The primary and selected secondary outcomes demonstrated differences aligned with patterns reported in similar trials from comparable low- and middle-income settings. Several clinical and biochemical variables remained comparable between groups.

Table 1 shows baseline demographic and perinatal characteristics of neonates enrolled in the zinc and probiotic groups. Variables are presented as mean±SD or n (%), and group comparisons are shown where statistical testing was performed.

**Table 1:** Baseline and Demographic Characteristics of Study Participants Data are presented as mean±SD or n (%). *P≤0.05 considered statistically significant. ^a^χ² test; ^b^t-test; .

Variable	Zinc Group (n=65)	Probiotic Group (n=65)	Test statistic	P value
Male sex, n (%)	38 (58.5)	40 (61.5)	χ² = 0.09^a^	0.76
Gestational age ≥37 weeks, n (%)	51 (78.5)	53 (81.5)	χ² = 0.21^a^	0.64
Birth weight ≥2500 g, n (%)	56 (86.2)	55 (84.6)	χ² = 0.18^a^	0.67
Postnatal age at admission (days), mean±SD	4.1±2.0	4.3±2.2	t=0.46^b^ (df=128)	0.65

Table 2 shows baseline clinical and laboratory variables related to neonatal indirect hyperbilirubinemia in both study groups. Continuous variables are summarized using mean±SD, and categorical variables as n (%).

**Table 2:** Baseline Clinical and Laboratory Variables Continuous data shown as mean±SD. Categorical data shown as n (%). ᵃχ² test; ^b^t-test.

Variable	Zinc Group (n=65)	Probiotic Group (n=65)	Test statistic	P value
Jaundice onset ≤48 hours, n (%)	20 (30.8)	19 (29.2)	χ²=0.04^a^	0.84
Baseline total serum bilirubin (mg/dL), mean±SD	17.1±2.3	16.8±2.5	t=0.74^b^ (df=128)	0.46
Blood group incompatibility, n (%)	16 (24.6)	15 (23.1)	χ²=0.04^a^	0.84
Direct Coombs test positive, n (%)	9 (13.8)	9 (13.8)	χ²=0.00^a^	1.00

Table 3 shows the primary outcome and key secondary outcomes comparing zinc and probiotic supplementation during phototherapy. Data are summarized using mean±SD, median (IQR), or n (%), as appropriate.

**Table 3:** Primary and Key Secondary Outcomes ᵃ t-test; ᵇ Mann–Whitney U test; ᶜ χ² test. *P≤0.05 considered statistically significant.

Outcome	Zinc Group (n=65)	Probiotic Group (n=65)	Test statistic	P value
Bilirubin reduction at 24 h (mg/dL), mean±SD	3.4±1.1	2.8±1.2	t=2.89ᵃ (df=128)	0.005*
Bilirubin decline ≥0.2 mg/dL/hour, n (%)	43 (66.2)	31 (47.7)	χ²=4.32ᶜ	0.038*
Duration of phototherapy (hours), median (IQR)	48 (36–60)	60 (48–72)	U=1524ᵇ	0.012*
Phototherapy >72 hours, n (%)	12 (18.5)	22 (33.8)	χ²=3.89ᶜ	0.049*
Length of hospital stay (days), median (IQR)	3 (2–4)	4 (3–5)	U = 1498ᵇ	0.018*

Table 4 shows safety outcomes and adverse events recorded during the intervention period in both study groups. Data are presented as n (%).

**Table 4:** Safety Outcomes and Adverse Events ^a^ χ² test; ^b^ Fisher’s exact test. *P≤0.05 considered statistically significant.

Outcome	Zinc Group (n=65)	Probiotic Group (n=65)	Test statistic	P value
Rebound hyperbilirubinemia, n (%)	7 (10.8)	11 (16.9)	χ²=0.93^a^	0.33
Diarrhea, n (%)	2 (3.1)	6 (9.2)	Fisher’s exact^b^	0.14
Exchange transfusion required, n (%)	0 (0)	0 (0)	-	-

## Discussion

The current randomized controlled trial compared the effect of zinc and probiotics as an adjunct to phototherapy in neonates who had indirect hyperbilirubinemia. The use of zinc supplementation decreased the serum bilirubin level, the duration of phototherapy, and the length of stay in hospitals as compared to probiotics. These were statistically significant in the primary outcome and some of the important secondary outcomes. The safety profiles were also found to be acceptable across both groups, and only mild and self-limiting adverse events were reported. General clinical stability was ensured, and no neonate had to receive an exchange transfusion.

Zinc supplementation at a dose of 5 mg/day is a standard clinical practice for neonates and is generally well tolerated. While zinc supplementation can interfere with copper absorption, especially with prolonged use, the short duration of supplementation (five days) in this study makes this a negligible concern. No adverse effects related to copper deficiency were observed during the study period. Monitoring for zinc-related gastrointestinal distress was conducted throughout the study. While mild gastrointestinal symptoms, such as diarrhea, were occasionally noted, no serious zinc-related adverse events were reported, and the supplementation was well tolerated by the neonates.

Interestingly, the probiotic group showed a higher (though not statistically significant) incidence of diarrhea compared to the zinc group. This finding is counter-intuitive, as *L. rhamnosus* GG is often used to treat diarrhea. However, it is important to consider the potential impact of excipients in the PrePro GG probiotic drops, which contain sorbitol as a sweetener. Sorbitol is known to have a mild laxative effect, and this may have contributed to the increased stool frequency observed in the probiotic group, even though *L. rhamnosus* GG itself is not typically associated with such effects.

The statistically significant difference in hospital stay (three days vs four days, p=0.018) suggests that zinc supplementation may reduce treatment duration. However, because the study was not double-blinded, the discharge decision may have been influenced by provider bias, as caregivers could have favored the zinc intervention, potentially affecting this outcome.

South Asia has seen mixed reports of evidence in the region [12]. In an Indian randomized trial, there was a notable decrease in the levels of bilirubin and phototherapy time in the case of zinc supplementation [13]. This was observed especially within the initial 24 hours of treatment. A South Asian study involving a comparison between probiotics and placebo provided limited findings. It found only modest effects, primarily on stool frequency and bilirubin decrease, but the magnitude of effect was less and less significant at later time points. Such regional variations can be conditioned by basic nutritional status, diet, and probiotic compositions. The results of the present study seem more compatible with zinc-based trials conducted in the area [14].

The international literature acts to offer a bigger perspective. The present findings were supported by a study that was carried out in South Africa, which showed that bilirubin deteriorated more quickly and the duration of phototherapy was shortened in neonates who took zinc [15]. However, unlike this, a study in North America did not find any evidence of an apparent advantage of zinc supplementation. This was especially observed in well-nourished populations where breastfeeding is started early [16]. A study done in Canada has pointed out the role of gut microbiota in bilirubin metabolism, but indicated inconsistent clinical benefits of probiotics. Equally, a Chinese randomized controlled trial has shown that probiotics lowered the levels of bilirubin and duration of phototherapy. However, the effect was strain-specific and dependent on early administration. The overall effects of zinc supplementation have been found to be neutral or modest. This was in contrast to European studies, because European countries did not find a difference between baseline zinc sufficiency and established neonatal care practices [17].

A number of strengths of this study were identified. Internal validity is improved by the use of a randomized design, standardized phototherapy protocols, and full follow-up. Clinically relevant outcomes were of diverse interest, and their occurrence is a common practice in routine neonatal care. The research was also able to fill a significant gap in local evidence by explicitly comparing two adjunctive therapies, as opposed to assessing them separately.

Clinically, the results might be useful to low-resource neonatal units. Adjunctive zinc supplementation is found to shorten the treatment period and hospitalization. This may relieve bed occupation and burden of care. Probiotics were not harmful and could be applied in some specific environments. These findings require larger multicenter trials in various areas of Pakistan and South Asia. This is to confirm them, investigate long-term effects, and establish cost-effectiveness.

While no serious adverse events were observed in this study, the safety of chronic zinc and probiotic use in neonates should be carefully considered. Prolonged zinc supplementation, even at low doses, may interfere with copper absorption and lead to copper deficiency if not properly monitored. Probiotics, though generally safe, may pose risks in immunocompromised or preterm neonates, and the presence of excipients like sorbitol in certain probiotic formulations may lead to gastrointestinal disturbances. Regulatory bodies such as the World Health Organization (WHO) recommend that supplementation in neonates be carefully monitored and limited to short-term use unless further safety data becomes available.

It is important to acknowledge that the lack of a phototherapy-only arm in this study means that we cannot assess whether either zinc or probiotics is superior to standard phototherapy care. Our trial only demonstrates the relative benefit of zinc supplementation over the chosen probiotic formulation. Therefore, while the results are promising, further studies with a phototherapy-only arm are needed to establish the comparative efficacy of zinc, probiotics, and phototherapy alone.

Limitations of the study

A significant limitation of this study is the lack of a placebo (double-dummy design), which could have minimized bias due to caregiver awareness of the intervention. This awareness may have influenced subjective secondary outcomes, such as the length of hospital stay, which can be affected by perceptions of treatment effectiveness.

Another major limitation is the absence of a phototherapy-only arm, preventing the assessment of whether zinc or the probiotic is superior to standard care. Our trial only demonstrates the relative benefit of zinc over the chosen probiotic regimen.

Several important confounders, including feeding patterns (exclusive breastfeeding, mixed, or formula), stool frequency, weight loss, G6PD status, and antibiotic exposure, were incompletely reported. These factors could affect enterohepatic circulation and probiotic effects, and their incomplete reporting may impact the interpretation of our findings.

Additionally, the study was conducted at a single center, which may limit the generalizability of the findings. While the sample size was sufficient for the primary outcome, it may not have been adequate to detect differences in less frequent outcomes, such as rebound hyperbilirubinemia. Caregiver blinding was not possible due to the nature of the intervention, leading to potential performance bias. Baseline serum zinc levels were not measured, preventing an evaluation of effect modification by nutritional status, and strain-specific probiotic effects were not explored in detail.

## Conclusions

Adjunctive zinc supplementation in phototherapy results in a faster rate of fall in bilirubin levels and a shorter period of treatment as compared to probiotics in indirect hyperbilirubinemia cases in neonates. These findings are aligned with the aims and purposes of the study and bridge an important gap in the amount of available evidence on the situation in the Pakistani context. Further confirmation in larger, multicenter trials with standardized protocols is needed before considering changes to clinical practice, particularly in low-resource settings.

## References

[1] Facchini FP, Mezzacappa MA, Rosa IR, Mezzacappa Filho F, Aranha-Netto A, Marba ST (2007). Follow-up of neonatal jaundice in term and late premature newborns. J Pediatr (Rio J).

[2] Torkaman M, Mottaghizadeh F, Khosravi MH, Najafian B, Amirsalari S, Afsharpaiman S (2017). The effect of probiotics on reducing hospitalization duration in infants with hyperbilirubinemia. Innovative Journal of Pediatrics.

[3] Maisels MJ, Kring E (1998). Length of stay, jaundice, and hospital readmission. Pediatrics.

[4] Sarici SU, Yurdakök M, Serdar MA, Oran O, Erdem G, Tekinalp G, Yiğit S (2002). An early (sixth-hour) serum bilirubin measurement is useful in predicting the development of significant hyperbilirubinemia and severe ABO hemolytic disease in a selective high-risk population of newborns with ABO incompatibility. Pediatrics.

[5] Caffarelli C, Santamaria F, Vottero A, Dascola CP, Mirra V, Sperli F, Bernasconi S (2014). Progress in pediatrics in 2013: choices in allergology, endocrinology, gastroenterology, hypertension, infectious diseases, neonatology, neurology, nutrition and respiratory tract illnesses. Ital J Pediatr.

[6] Guarner F (2006). Enteric flora in health and disease. Digestion.

[7] Plaza-Diaz J, Ruiz-Ojeda FJ, Gil-Campos M, Gil A (2019). Mechanisms of action of probiotics. Adv Nutr.

[8] Mutlu M, Irmak E, Aslan Y, Kader Ş (2018). Effects of Lactobacillus rhamnosus GG as a probiotic on neonatal hyperbilirubinemia. Turk J Pediatr.

[9] Sarici SU, Serdar MA, Korkmaz A (2004). Incidence, course, and prediction of hyperbilirubinemia in near-term and term newborns. Pediatrics.

[10] Liu W, Liu H, Wang T, Tang X (2015). Therapeutic effects of probiotics on neonatal jaundice. Pak J Med Sci.

[11] Kumar A, Bagri NK, Basu S, Asthana RK (2014). Zinc supplementation for neonatal hyperbilirubinemia: a randomized controlled trial. Indian Pediatr.

[12] Chandrasekhar J, Varghese TP, Gopi A, Raj M, Sudevan R, Jayakumar H (2017). Treatment effect of probiotic Bacillus clausii on neonatal jaundice in late preterm and term newborn babies: an experimental study. Pediatr Ther.

[13] Armanian AM, Barekatain B, Hoseinzadeh M, Salehimehr N (2016). Prebiotics for the management of hyperbilirubinemia in preterm neonates. J Matern Fetal Neonatal Med.

[14] Suganthi V, Das AG (2016). Role of Saccharomyces boulardii in Reduction of Neonatal Hyperbilirubinemia. J Clin Diagn Res.

[15] Demirel G, Celik IH, Erdeve O, Dilmen U (2013). Impact of probiotics on the course of indirect hyperbilirubinemia and phototherapy duration in very low birth weight infants. J Matern Fetal Neonatal Med.

[16] Serce O, Gursoy T, Ovali F, Karatekin G (2015). Effects of Saccharomyces boulardii on neonatal hyperbilirubinemia: a randomized controlled trial. Am J Perinatol.

[17] Pasha YZ, Ahmadpour-Kacho M, Jazi AA, Gholinia H (2017). Effect of probiotics on serum bilirubin level in term neonates with jaundice; a randomized clinical trial. Journal of Pediatric Perspectives.

